# Carbon Formation during Methane Dry Reforming over Ni-Containing Ceria-Zirconia Catalysts

**DOI:** 10.3390/nano12203676

**Published:** 2022-10-19

**Authors:** Ekaterina Smal, Yulia Bespalko, Marina Arapova, Valeria Fedorova, Konstantin Valeev, Nikita Eremeev, Ekaterina Sadovskaya, Tamara Krieger, Tatiana Glazneva, Vladislav Sadykov, Mikhail Simonov

**Affiliations:** Department of Heterogeneous Catalysis, Boreskov Institute of Catalysis, 630090 Novosibirsk, Russia

**Keywords:** methane dry reforming, carbon formation, supercritical synthesis, fluorite, heterogeneous catalysis

## Abstract

Two series of Ni/Ce(Ti/Nb)ZrO_2_ catalysts were prepared using citrate route and original solvothermal continuous flow synthesis in supercritical isopropanol and studied in dry reforming of methane (DRM). TEM, XPS and FTIRS of adsorbed CO confirm influence of support composition and preparation method on the catalysts’ morphology and surface features. The oxygen mobility was studied by isotope heteroexchange with C^18^O_2_. After testing in DRM, carbon deposits after catalysts’ testing in DRM were investigated by temperature-programmed oxidation with thermo-gravimetric analysis. The lowest amounts of carbon deposits were obtained for unmodified Ni-CeZr and Ni-CeNbZr compositions. Ti addition lead to an increased amount of carbon, which was removed at higher temperatures. The use of supercritical supports also resulted in the formation of a higher amount of coke. Catalysts prepared by the supercritical synthesis were tested in DRM for 25 h. The highest activity drop was observed in the first three hours. For all compositions, close values of carbon deposits were revealed.

## 1. Introduction

In recent years, dry reforming of methane (DRM) (Equation (1)) is widely investigated because it allows carrying out processes of biogas utilization, conversion of natural gas with a high CO_2_ content and utilization of such greenhouse gases as methane and carbon dioxide [1,2]. Stoichiometrically, it produces industrially important synthesis gas with a H_2_/CO = 1 ratio, which can be a feedstock for the production of valuable long-chain hydrocarbons by Fischer-Tropsch process [3,4]:

CH_4_ + CO_2_ → 2CO + 2H_2_      ΔH^0^_298K_ = 260.5 kJ/mol (1)

According to thermodynamic calculations, H_2_/CO ratio decreases with increasing either temperature or CO_2_/CH_4_ ratio [5]. In addition, the H_2_/CO ratio can be changed by simultaneous occurrence of side reactions. The reverse water–gas shift reaction leads to hydrogen consumption and CO formation and decreases the H_2_/CO ratio [6,7]:

CO_2_ + H_2_ → CO + H_2_O      ΔH^0^_298K_ = 41.0 kJ/mol (2)

Addition of such reagents as H_2_O, CO_2_ and O_2_ in different combinations can be used to control the H_2_/CO ratio [4].

Despite the above-mentioned benefits, dry reforming of methane has not yet found industrial application. The main disadvantage of this process is rapid catalyst deactivation due to carbon deposition on catalysts surface and sintering of both metal and support particles at high reaction temperatures [8]. 

The carbon formation can occur by reactions of methane decomposition (Equation (3)) and CO disproportionation (or Boudouard reaction) (Equation (4)) [9,10]:

CH_4_ → C + 2H_2_      ΔH^0^_298K_ = 75.0 kJ/mol (3)

2CO → C + CO_2_      ΔH^0^_298K_ = −172.0 kJ/mol (4)

The reaction of methane cracking is endothermic and can proceed at a significant rate above 557 °C, while the Boudouard reaction is exothermic, occurring below 700 °C. So, carbon can be formed by both these reactions in the temperature range of 557–700 °C [11,12]. In the work [13], the operating conditions required to prevent carbon deposition at different pressures (0.6, 1, 10 atm) and gas feed ratios (CO_2_/CH_4_) were determined by thermodynamic analysis. It was shown that when a reaction was carried out at 1 atm and CO_2_/CH_4_ = 1, carbon formation was possible at temperatures lower than 870 °C. 

Different types of carbon can be formed during the methane dry reforming depending on the reaction conditions and catalyst composition [14]. In general, amorphous carbon is known to be more reactive and easily gasified, while graphitic carbon is oxidized at higher temperatures [15,16,17,18].

Formation of different types of carbon with different reactivity were demonstrated in several works [8,19,20,21]. 

At least two types of carbon deposits were identified on Ni/SiO_2_ in [19]: the first type of carbon was formed at the beginning of the catalytic test and oxidized at low temperature; the second type of carbon was more stable towards oxidation and its accumulation results in catalyst deactivation.

For Ni/Al_2_O_3_ catalyst, three types of carbonaceous species were observed being oxidized at 150–220 °C (C_α_), 530–600 °C (C_β_) and > 650 °C (C_γ_) [20]. The C_β_ and C_γ_ were formed at low (500–600 °C) reaction temperatures. At reaction temperatures above 750 °C the main form was C_α_. The active C_α_ species were assumed to take part in CO formation, while the less active C_β_ and C_γ_ participated in catalyst deactivation. At high reaction temperature C_β_ and C_γ_ were removed that can be a reason for lower deactivation rate and higher catalyst stability. The similar correlations were also obtained in the following work. The temperature-programmed hydrogenation of Ni/MgO catalysts after DRM showed the presence of two carbonaceous forms being removed at 280–430 °C (C_α_) and above 600 °C (C_β_), respectively [21]. The intensity of C_β_ peak increased significantly with the time on stream. It also was suggested that C_α_ is the reaction intermediate, while C_β_ formation leads to catalysts’ deactivation.

Catalytic activity and stability to carbonization depend on many factors, such as type of active metal, preparation method, nature of precursors, calcination temperature, reduction pretreatment and support nature [22]. Noble-metal catalysts provide high catalytic activity and stability to coke formation [23]. However, their industrial application is impossible due to their high cost. Ni-containing catalysts are a suitable alternative because they provide a high catalytic activity and have affordable price. The main drawback of such catalysts is their deactivation due to coke deposition [14]. 

A possible way to minimize carbon formation is utilization of oxide supports with a high oxygen storage capacity (OSC) [4,24]. Participation of support oxygen in the gasification of coke precursors should improve catalysts’ stability to coking. Ceria is widely investigated as a catalyst support due to its high OSC and Ce^4+^/Ce^3+^ redox potential. It is also known to resist sintering and exhibit strong metal–support interaction, leading to a high metal dispersion [1].

CeO_2_ modification by Zr leads to improved textural features, thermal stability, reducibility and oxygen storage/transport properties [16,25,26]. The introduction of other dopants such as W, La or Pr also enhances these ceria properties [8,27,28,29]. In our previous works [30,31,32], we have shown that the introduction of titanium and niobium increases the number of oxygen vacancies in the structure of ceria–zirconia.

The carbon formation by CH_4_ decomposition is a structure-sensitive reaction, so it can be influenced by modification of the catalyst surface [14]. A high dispersion of Ni particles should reduce the formation of carbon deposits [33,34], since the metal atom ensembles necessary for carbon formation are larger than those needed for CH_4_ reforming [35].

The preparation method as well as method of nickel addition has a strong influence on the catalysts’ morphology and surface features [36].

As an example, Ni-containing ceria–zirconia catalysts Ni/Ce_0.83_Zr_0.17_O_2_ were prepared by four different techniques including two impregnation methods—dry impregnation and strong electrostatic adsorption, and two one-pot routes—co-precipitation and combustion synthesis [37]. According to CO chemisorption data, Ni dispersion was the highest for sample prepared by the adsorption method. The same catalyst showed the highest initial methane conversion, while the other three samples demonstrated close results despite the different values of nickel dispersion. During TGA oxidation tests after reaction, the catalyst prepared by adsorption showed the highest weight loss. 

A similar situation was shown by Makri et al. [8], when amount of carbon deposits was maximal for the most active composition 5%Ni/Ce_0.8_Pr_0.2_O_2_. 

In our previous work [30], we have investigated Ni-containing catalysts based on ceria–zirconia (also doped by Ti and Nb cations) in methane dry reforming reaction. It was devoted to the study of textural and structural features and reducibility of supports and catalysts depending on preparation method and its effect on catalytic activity.

Catalysts’ supports were obtained by two preparation techniques—by traditional citrate route and by original solvothermal continuous flow synthesis in supercritical alcohols. Synthesis in supercritical isopropanol allows obtaining single-phased oxides with more uniform distribution of components. Ti and Nb incorporation leads to increased amounts of oxygen vacancies and to higher catalytic activity. Moreover, it was shown that catalytic activity depends on both support composition and preparation method. 

In this article, we continue our research, and the purpose of this work was to find out how the modification of the support chemical composition affects the carbon formation in a methane dry reforming reaction and how formed carbon affects the catalytic activity. To achieve this goal, the surface features of the catalysts were investigated in detail using XPS and FTIR spectroscopy of the adsorbed CO. Long-term stability tests in DRM were carried out to check the catalysts’ stability and the amount of formed carbon was determined by TPO-TGA with mass-spectroscopic analysis. Since the coking stability of catalysts is strongly related to the support’s oxygen mobility, the oxygen diffusion coefficients were estimated using the temperature-programmed isotope exchange of oxygen with C^18^O_2_. The effect of titanium and niobium addition on the oxygen mobility and carbonization stability of Ni-containing ceria–zirconia catalysts was studied for the first time.

## 2. Materials and Methods

### 2.1. Catalysts’ Preparation

The first series of supports was prepared by citrate methods. Ce_0.75_Zr_0.25_O_2_ and Ce_0.75_Nb_0.1_Zr_0.15_O_2_ oxides were prepared by Pechini route [31] using citric acid (CA) and ethylene glycol (EG) as polyester complexing agents. The molar ratio of CA:EG:Me (metals) was 3.75:11.25:1. Citric acid was dissolved in ethylene glycol, then corresponding salts were added to this solution. The mixture was stirred for 2 h and then heated at 80 °C to evaporate water. The resulting dried gel was calcined at 700 °C for 2 h in air, obtaining oxide powder. Ce_0.75_Ti_0.1_Zr_0.15_O_2_ and Ce_0.75_Ti_0.05_Nb_0.05_Zr_0.15_O_2_ oxides were prepared by citrate method in which aqueous solution of citric acid was used; CA:(metals) ratio was 5:1. The further procedure is the same as described above. 

The second series of supports with the same compositions was prepared by the continuous flow solvothermal synthesis using supercritical isopropanol by technique described earlier [30,31]. A detailed installation scheme is presented in [31]. The resulting precipitate was dried at 200 °C and calcined at 700 °C for 2 h in air.

Catalysts with 5 wt.% Ni were prepared by the incipient wetness impregnation of obtained oxides with water solution of Ni(NO_3_)_2_ followed by drying and calcination at 700 °C for 2 h. Catalysts’ compositions and abbreviations are presented in the Table 1.

### 2.2. Catalysts’ Characterization

Experiments of the low-temperature adsorption/desorption of nitrogen were carried out using a Quadrasorb evo (Quantachrome Instruments, Boynton Beach, Florida, USA) installation. Specific surface area of samples was determined by BET method. The desorption branch of isotherm was used to estimate the pore volumes by the BJH method.

A Bruker D8 Advance diffractometer (Bruker, Germany) with CuKα radiation and a position sensitive detector LynxEye was used to record XRD patterns with step 0.05° in 2θ scanning range 10–85°. Parameter calculations were performed by the Rietveld method using Bruker TOPAS software.

TEM (transmission electron microscopy) micrographs were obtained with Themis-Z 3.1 instrument (TFS, Waltham, MA, USA) equipped with X-FEG-monochromator and CS/S double corrector, accelerating voltage 200 kV and with JEM-2200FS transmission electron microscope (JEOL Ltd., Akishima, Tokyo, Japan, acceleration voltage 200 kV, lattice resolution~1Å) equipped with a Cs-corrector. Elemental analysis was performed with Super-X EDS detector (energy resolution about 120 eV) in HAADF-STEM mode. 

A photoelectron spectrometer (SPECS Surface Nano Analysis GmbH, Berlin, Germany) with a hemispherical electron energy analyzer PHOIBOS-150 and X-ray source XR-50 with a double Al/Mg anode was used for recording XPS spectra. The spectra were obtained using the non-monochromatized AlKα radiation (hν = 1486.61 eV). The powders of catalysts were pressed into a pellet, which was fixed on a sample holder using a double-sided conductive adhesive tape. The binding energy (BE) scale was calibrated by the Ce*3d*_3/2_-u’’’ cerium line (BE = 916.7 eV). For estimation of relative elements concentrations in surface layers, XPS lines with the photoionization cross-sections of the corresponding terms were taken into account [38]. From each spectrum, a Shirley-type background was subtracted, then the experimental curve was deconvoluted into a number of lines corresponding to the photoemission of electrons from atoms in different chemical environments. The CasaXPS software was used for the curve fitting. The line shape was approximated by a symmetric function obtained by multiplying the Gauss and Lorentz functions. Before recording the spectra, the samples were pretreated in a high-pressure cell in a hydrogen atmosphere at 450 °C for 30 min.

A Shimadzu IRTracer-100 (Shimadzu, Kyoto, Japan) spectrometer was used for recording FTIR spectra. The spectra of adsorbed CO at −196 °C and at room temperature were obtained in the 400–6000 cm^−1^ range, accumulating 200 scans at 4 cm^−1^ resolution. The samples were pressed into pellets with size of 1 × 2 cm^2^. A sample placed into the IR cell was heated in a vacuum to 600 °C and then calcined in a hydrogen atmosphere (100 Torr of H_2_) for 1 h at this temperature. After that, the sample was evacuated to a residual gas pressure of at least 10^−4^ Torr and cooled to room temperature. CO was adsorbed at −196 °C and CO pressure from 0.1 to 10 Torr. After obtaining the spectra at liquid nitrogen temperature, the sample was heated to room temperature and corresponding spectra were recorded. The obtained spectra were normalized to the optical thickness of the pellets. To obtain differential spectra, the spectrum before CO adsorption was subtracted from the spectrum after adsorption. The analysis of IR spectra was carried out by decomposition of the corresponding IR bands into individual Gaussian components. The amount of surface sites of different types were estimated using the integral absorption coefficients from the integrated intensities of the characteristic absorption bands [39]. The error in measuring the sites amount was 20%.

The oxygen mobility of samples was investigated by temperature-programmed isotope exchange (TPIE) of oxygen with C^18^O_2_. The experiments were carried out with the sample weight of 50 mg placed into flow quartz tube reactor with the inner diameter of 3 mm. The samples were preliminarily pretreated in He + 1% O_2_ flow (flow rate 25 mL/min) at 700 °C for 30 min and cooled down to room temperature in He + 1% CO_2_ flow (flow rate 25 mL/min). After the steady state was reached, the feed gas mixture was switched to the mixture of the same composition but containing ^18^O label. The temperature was raised from 50 °C to 700 °C with a ramp of 5 °C/min. The effluent gases were analyzed by the UGA 200 mass spectrometer (Stanford Research Systems, Sunnyvale, CA, USA). The analysis of ^18^O atomic fraction (*α*) and C^16^O^18^O molecular fraction (*f*_16-18_) responses was made for calculating the values of the heteroexchange rate (*R*), oxygen tracer diffusion coefficient (D^*^) and their effective activation energies (*E_a,R_*, *E_a,D_*) using a mathematical model (Supplementary Materials) [40,41].

To estimate the amount of carbon formed during catalytic tests in DRM reaction, the temperature-programmed oxidation coupled with thermogravimetric analysis (TPO-TGA) with synchronous analysis of the gas phase was used. Thermal analysis was carried out using a STA 409 PC instrument (NETZSCH, Selb, Germany). The Ni/Ce-Zr samples after DRM reaction were placed into corundum crucibles. The weight of the sample was ca. 75 mg. The oxidizing mixture contains O_2_ (10% vol.)/He with the feeding rate 30 mL/min. Differential thermal analysis (DTA) curves were recorded in the temperature range from 50 to 800 °C with 10 °C/min heating rate. Effluent gases concentrations were measured by UGA 200 gas analyzer (Stanford research systems, Sunnyvale, CA, USA).

### 2.3. Catalysts’ Testing in Methane Dry Reforming

The catalysts’ activity in methane dry reforming reaction was investigated by tests in a tubular quartz plug flow reactor with the feed mixture of 15% CH_4_ + 15% CO_2_ + N_2_ balance in the temperature range 600–750 °C and contact time 10 ms. The catalyst weight was diluted with quartz in 1:1 ratio (grains size 0.5–0.25 mm). The variation of temperature was realized stepwise by 50 °C, analysis of products concentration at each step was recorded during 30 min. The preliminary reduction treatment of catalysts was made in a stream of 5% H_2_ in He at 600 °C for an hour. The gas analyzer with IR sensors for CO, CO_2_ and CH_4_, and electrochemical sensor for H_2_ (Boner LLC, Novosibirsk, Russia) was used for the reaction mixture analysis.

Long-term stability tests were carried out for 25 h at 700 °C with the same catalyst pretreatment and reaction feed of 15% CH_4_ + 15% CO_2_ + Ar. 

## 3. Results and Discussion

### 3.1. Characterization of Fresh Catalysts

#### 3.1.1. Study of Structure and Texture

According to XRD data, all mixed oxides are ceria–zirconia solid solutions with the cubic fluorite structure (PDF 81–0792). The absence of peaks corresponding to Ti or Nb oxides, as well as changing the lattice parameter of fluorite phase [30] after their addition, suggests the incorporation of doping cations into the ceria–zirconia mixed oxide structure. In the case of citrate samples, small amounts of zirconium oxide ZrO_2_ impurity are observed for samples doped by Ti since titanium cations can be incorporated into the fluorite lattice instead of zirconium and displace it. Synthesis in supercritical media provides more uniform distribution of components, and no ZrO_2_ impurities are observed for them. More detailed information and diffraction patters of obtained samples are given in our earlier work [30]. 

Table 2 presents catalyst’s textural characteristics and crystallite sizes calculated from XRD data. Crystallites size of fluorite phase of citrate samples is ~11 nm and does not depend on the sample’s chemical composition. Crystallites size of samples prepared by supercritical synthesis is more sensitive to the oxide composition and varies in the range of 9–15 nm.

Crystallites size of nickel oxide in the citrate series is in the range of 18–24 nm and in the supercritical series—within 20–30 nm. Apparently, a higher NiO dispersion in citrate samples is related to their higher specific surface area. Pore volume of samples slightly depends on the method of preparation and increases with the introduction of Ti and Nb.

#### 3.1.2. Study of Surface Properties by XPS

The chemical states and relative concentrations of elements in the (sub)surface layers of catalysts were studied by the X-ray photoelectron spectroscopy. To obtain the surface state close to that under reaction conditions, the samples were pretreated in a hydrogen atmosphere before recording the spectra.

**Figure 1:**
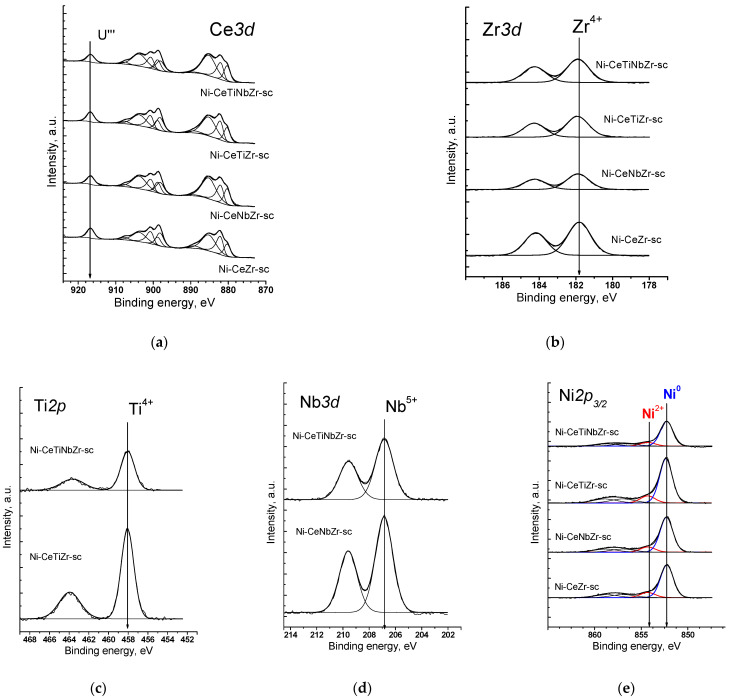
XPS spectra of catalysts prepared by supercritical synthesis after reduction treatment in H_2_ at 450 °C: (**a**) Ce*3d* spectra, (**b**) Zr*3d* spectra, (**c**) Ti*2p* spectra, (**d**) Nb*3d* spectra, (**e**) Ni*2p*_3/2_ spectra.

Figure 1a shows the Ce*3d* spectra of catalysts prepared by supercritical synthesis. Cerium is in the Ce^3+^ and Ce^4+^ states. It is well known that Ce*3d* spectra have a complex shape. As a result of the spin–orbit interaction, the cerium 3d level splits into two sublevels Ce*3d*_5/2_ and Ce*3d*_3/2_, which leads to appearance in the XPS spectrum of doublet with integral line intensities in 3:2 ratio. Each component of the doublet splits into three lines in the case of CeO_2_ (v/u, v’’/u’’, v’’’/u’’’) or into two lines in the case of Ce_2_O_3_ (v’/u’, v_0_/u_0_). The fraction of Ce^3+^ ions can be estimated from relative intensities of these components [42]. In accordance with the results of spectra deconvolution into individual components, the proportion of Ce^3+^ ions is 60–70% for citrate samples and 50–60% for supercritical samples (Table 3).

The spectrum of Zr*3d* is a Zr*3d*_5/2_-Zr*3d*_3/2_ doublet with integral intensities of the components in ratio of 3:2 (Figure 1b). The spin–orbit splitting (the difference between the binding energies of Zr*3d*_5/2_ and Zr*3d*_3/2_) is 2.39 eV. All Zr*3d* spectra are well described by one doublet. The binding energy of Zr*3d*_5/2_ (181.8 eV) corresponds to zirconium in the Zr^4+^ state. For stoichiometric oxide ZrO_2_, the value of the binding energy Zr*3d*_5/2_ lies in the range of 182.2–183.3 eV [43,44].

The Ti*2p* spectra are shown in Figure 1c. The 2p level of titanium splits into two sublevels Ti*2p*_3/2_ and Ti*2p*_1/2_ due to spin–orbit interaction, the spin–orbit splitting is 5.66 eV. The Ti*2p*_3/2_ peak has a symmetric shape with the Ti*2p*_3/2_ binding energy being 458.2 eV, which corresponds to titanium in the Ti^4+^ state. In the literature, the values of the binding energy Ti*2p*_3/2_ for TiO_2_ are given in the range of 458.7–459.2 eV [45,46].

The spectrum of Nb*3d* is Nb*3d*_5/2_—Nb*3d*_3/2_ doublet with the integral intensities of the components in 3:2 ratio (Figure 1d). The spin–orbit splitting (the difference between the binding energies of Nb*3d*_5/2_ and Nb*3d*_3/2_) is 2.8 eV. All Nb*3d* spectra are well described by one doublet. The binding energy of Nb*3d*_5/2_ varies in the range of 206.8–206.9 eV, which corresponds to niobium in the Nb^5+^ state. For stoichiometric oxide Nb_2_O_5_, the binding energy of Nb*3d*_5/2_ lies in the range of 206.2–207.9 eV [47,48]. 

In the Ni*2p*_3/2_ spectra of all samples (Figure 1e), there is a main intense asymmetric peak with a binding energy in the region of 852.3–852.4 eV, which corresponds to nickel in the Ni^0^ state [49]. The spectra also show a low-intensity peak in the range of 854.3 eV, which indicates the presence of a small amount of nickel atoms (~10%) in the Ni^2+^ state. There are also shake-up satellites in the range of 858.5 and 855.9 eV which are determined by multi-electron processes [50].

Table 3 presents data of elements atomic ratios. The preparation method apparently has a strong influence on the surface composition. The nominal Ce content is 0.75. On the surface of citrate samples, cerium content is less than the stoichiometric one, while the surface of supercritical samples is enriched by cerium. The introduction of doping cations has an ambiguous effect on the total cerium content on the surface. However, doping results in an increase in the [Ce^3+^]/[Ce] ratio regardless of the preparation method. It is in a good agreement with a higher amount of oxygen vacancies estimated from the XRD data, as was shown earlier in [30], since it is known that Ce^3+^ fraction correlates with the amount of oxygen vacancies [51]. A higher percentage of Ce^3+^ for samples prepared by citrate method may be associated with a lower total cerium content in the (sub)surface layers of oxide. 

In work [52], it was shown that the number of surface active sites, which are able to activate and transfer oxygen species, is proportional to Ce^3+^ content. Moreover, it can also increase Ni resistance to thermal sintering due to formation of [O^δ−, δ+^] dipolar layer on the surface of Ni particles and concomitant repulsive electrostatic forces between particles [17]. 

The surface concentration of Ni species is higher for catalysts prepared by supercritical synthesis. For citrate samples, Ni amount is lower than the nominal one ([Ni]/[Ce] + [Zr] + [Ti] + [Nb] = 0.14) which can be explained by decoration of Ni nanoparticles by support oxide layer. For both series of catalysts, the surface Ni content increases with addition of Ti and Nb separately, but decreases with their co-doping. 

#### 3.1.3. Study of Surface Sites by FTIR Spectroscopy of Adsorbed CO

**Figure 2:**
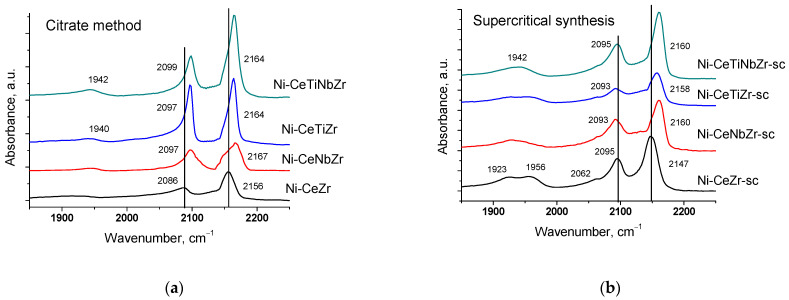
Differential FTIR spectra of CO adsorbed on catalysts prepared by citrate method (**a**) and by supercritical synthesis (**b**). pCO 10 Torr, T −196 °C.

The surface properties of catalysts preliminarily reduced in hydrogen were studied by IR spectroscopy of adsorbed CO. All spectra (Figure 2) contain absorption bands at ~2150–2160 cm^−1^ corresponding to OH-groups and carbonyl complexes of Ce^4+^ cations and a weak shoulder in the region ~2120–2130 cm^−1^ due to complexes with Ce^3+^ cations [53,54,55]. Such low intensity of Ce^3+^–CO bands suggests much lower surface concentration of adsorption sites of this type.

Incorporation of Ti and Nb cations into the ceria–zirconia lattice leads to a shift of the carbonyl band to higher frequency. It can be assigned to increase in the adsorption site strength, namely increasing Me^4+^–CO binding energy and at the same time decreasing the C-O bonding strength in the adsorbed species [8,56]. Low frequency bands at 2085–2095 and 1900–1957 cm^−1^ correspond to CO complexes with Ni^0^ sites [57,58]. 

**Figure 3:**
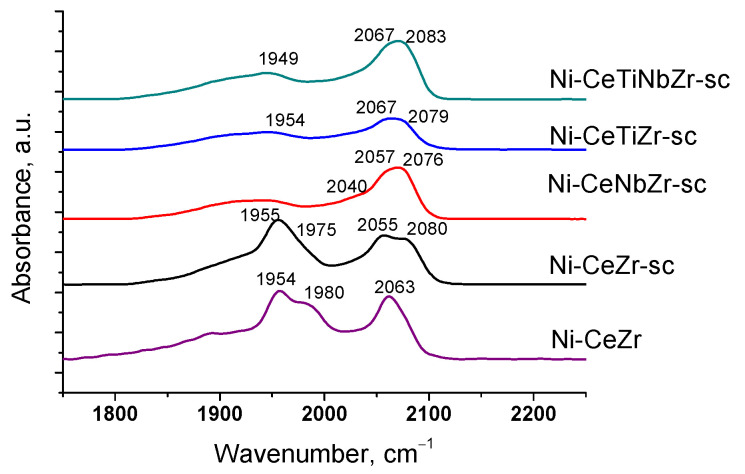
Differential FTIR spectra of CO adsorbed on catalysts at CO pressure 10 Torr and room temperature.

After heating samples from −196 °C to room temperature (Figure 3), bands corresponding to CO complexes with support cations disappear due to a weak bonding strength of such complexes [55]. Absorption bands at 1839–1980 and 2027–2083 cm^−1^ are assigned to bridging and terminal carbonyls formed on the surface of Ni^0^ particles, respectively [57,58]. Concentrations of various types of surface carbonyls estimated from the spectra at room temperature are presented in the Table 4. 

For undoped Ni-CeZr and Ni-CeZr-sc samples, high intensity bands of bridging carbonyls are observed. It is known that bridging carbonyls can be reactive in the Boudouard reaction, so existence of such sites can lead to carbon formation during dry reforming of methane [6]. In the case of supercritical series, introduction of Ti and Nb leads to decrease in amount of bridging carbonyls possibly due to decoration of metal particles by support fragments. For the doped citrate samples, no CO adsorption bands were observed in the spectra at room temperature, which may also be related to decoration of nickel particles by support oxide species.

#### 3.1.4. Study of Oxygen Mobility by Isotope Exchange with C^18^O_2_

**Figure 4:**
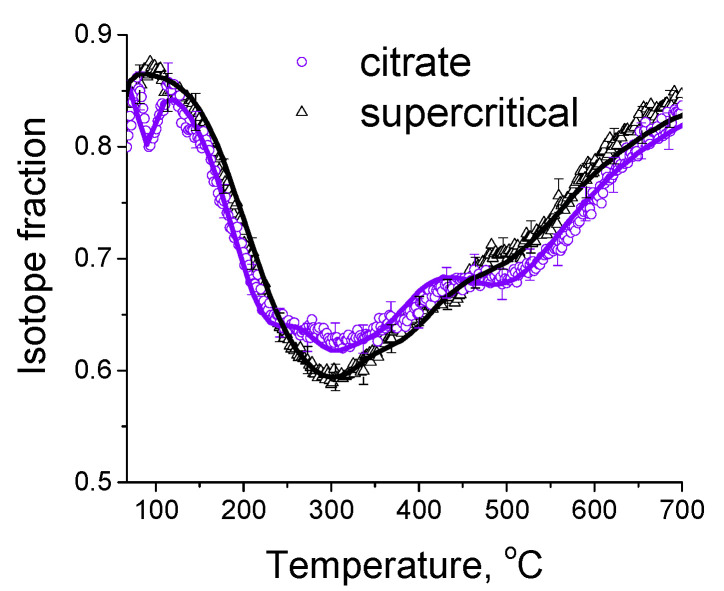
The TPIE curves for Ce_0.75_Zr_0.25_O_2_ samples synthesized by citrate method and by supercritical synthesis. Points—experiment, lines—modelling.

In order to investigate the relationship between the catalytic activity and oxygen transport properties, the oxygen isotope exchange studies were carried out. To elucidate the effect of synthesis technique on oxygen transport properties, a comparison of initial oxide Ce_0.75_Zr_0.25_O_2_ samples synthesized by citrate route and by supercritical synthesis was carried out by the temperature-programmed isotope heteroexchange (TPIE) of oxygen with C^18^O_2_. The TPIE curves are presented in the Figure 4. The amount of oxygen substituted during TPIE run almost reached overall oxygen amount in the samples, i.e., almost all oxygen in oxide samples is involved in the isotope exchange. The small low-temperature (below 100 °C) minimum observed for the citrate sample is apparently related to the exchange of weakly bound surface oxygen. The rest part of TPIE curves is similar for both samples and can be characterized by a set of overlapping peaks, making evidence of non-uniformity of oxygen diffusivity in the sample bulk. Moreover, the oxygen is more non-uniform in the case of citrate sample, which is evidenced by two explicit peaks at 300 and 500 °C, while the second peak is weakly expressed for supercritical sample.

To describe non-uniform oxygen diffusivity in such fluorite samples, two parameters have been selected: weighted average tracer diffusion coefficient calculated according to the Equation (5):

(*D*/*L*^2^)*_over_* = Σ(*θ_i_* *D/*L*^2^*_i_*)/100 (5)

*θ_i_* is fraction of oxygen with tracer diffusion coefficient (*D*/*L*^2^*_i_*), along with a non-uniformity coefficient (*β*) determined as a ratio of the average tracer diffusion coefficient of 50% fraction of oxygen substituting relatively fast to the average tracer diffusion coefficient of the rest fraction of oxygen (Supplementary Materials).

In this case, (*D*/*L*^2^)*_over_* values were 5.2∙10^−3^ s^−1^ and 2.2∙10^−3^ s^−1^ at 600 °C, non-uniformity coefficient *β* values were 38 and 13 for citrate and supercritical samples, respectively. While recalculating *D_over_* values assuming diffusion path length for spherical particles of *L* = 3*V*/*S* (where *S* is surface area, *V* is volume), these values almost match for both samples (Table 5). Hence, it can be concluded that using the technique of synthesis in supercritical media almost does not affect the average tracer diffusion coefficient, but leads to significant increase in uniformity of sample bulk oxygen diffusivity. 

Based on these conclusions, samples prepared in supercritical synthesis are more engaging and were selected for further investigation by isotope exchange. Since oxygen mobility is provided by the support, both initial oxides and catalysts were investigated to determine the effect of supported nickel on oxygen mobility.

**Figure 5:**
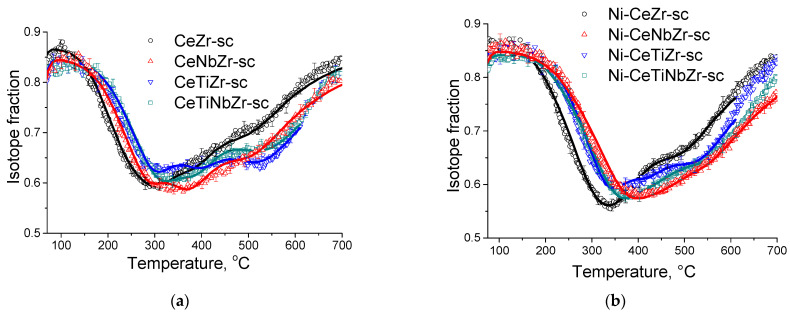
The TPIE curves for supports (**a**) and catalysts (**b**) prepared by supercritical synthesis.

Figure 5a demonstrates temperature dependencies for ^18^O isotope fraction in CO_2_ observed during TPIE run for supports synthesized in supercritical media. TPIE curves for all samples are also characterized by a set of overlapping peaks. By using the method described above, the average weighted oxygen tracer diffusion coefficient and non-uniformity coefficient values were calculated. The estimations obtained are given in the Table 5. The following conclusions can be made based on the estimations obtained. 

In contrast to expectations, while substituting 40% of Zr by Ti or by Ti + Nb, the bulk mean oxygen self-diffusion rate decreases by several times compared to Ce_0.75_Zr_0.25_O_2_. At the same time, non-uniformity of the sample bulk oxygen increases. It can be noted that the oxygen mobility, while substituting Zr by Ti + Nb, is higher compared to that for sample doped only by Ti. After substituting 40% of Zr by Nb, oxygen mobility and uniformity of oxygen diffusivity increase.

It should be noted that there is no unambiguous correlation between the number of oxygen vacancies estimated earlier in [30] and the coefficient of bulk oxygen mobility. This is not a contradiction since for mixed ceria–zirconia oxides the oxygen mobility is determined not only by the anion vacancies, but is defined by more complex set of defects and rearrangement of cations coordination spheres due to doping [59].

TPIE curves for the Ni-containing catalysts shift towards the higher temperature region compared to those for supports, which makes evidence of slowing the rate of oxygen isotope substitution in the samples (Figure 5b). Average weighted oxygen tracer diffusion coefficient *D_over_* and non-uniformity coefficient *β* significantly decrease after Ni deposition. It is to be noted that for such a high specific surface area of the samples and, hence, small diffusion path length (1–2 nm), near-surface effects can significantly affect the rate of ^18^O isotope diffusion into the sample bulk. In this case, the nickel effect is probably related to decreasing the oxygen permeability of the near-surface layer in the support. As a result of limiting effect of the near-surface layer, the fastest component of diffusivity is blocked; hence, TPIE curves become smoother, indicating higher homogeneity of support oxygen.

### 3.2. Catalytic Studies in Methane Dry Reforming

Data of catalytic thermal cycles were published earlier in [30]. However, they are reproduced here for the convenience of the reader. Table 6 presents steady-state methane conversions in methane dry reforming during the thermal cycle at 600–750–600 °C. Graphical data of methane conversion variations are given in Supplementary Materials (Figure S1). Conversions of CH_4_ and CO_2_ (not shown) increase with the temperature. To check the thermal stability of catalysts, the maximum temperature during the reaction was selected to exceed their calcination temperature. The drop in catalyst activity during temperature decrease after overheating can be related to sintering of the active component or with the carbon formation on the catalyst surface and blocking of active sites.

In the series of citrate samples, the highest methane conversion at 750 °C is achieved over unmodified Ni-CeZr catalyst and reached 48%. According to the values of conversion at 750 °C, activity increases in the row: Ni-CeTiNbZr < Ni-CeTiZr = Ni-CeNbZr < Ni-CeZr. It should be noted that methane conversions on the Ni-CeTiNbZr sample at the cooling step are very close to those exhibited in the heating step, suggesting a high stability of this catalyst despite its low activity.

The highest activity among both series of samples was obtained for the supercritical Ni-CeTiNbZr-sc catalyst. The methane conversion over this catalyst at 750 °C reached 51%. The conversion at maximum temperature increases in the series Ni-CeNbZr-sc < Ni-CeTiZr-sc < Ni-CeZr-sc < Ni-CeTiNbZr-sc. The lowest activity drop during thermal cycle is observed for the Ni-CeTiZr-sc composition.

More detailed description of catalytic tests and kinetics is given in our earlier works [30,31].

### 3.3. Characterization of Spent Catalysts

#### 3.3.1. Structural and Textural Studies of Spent Catalysts

**Figure 6:**
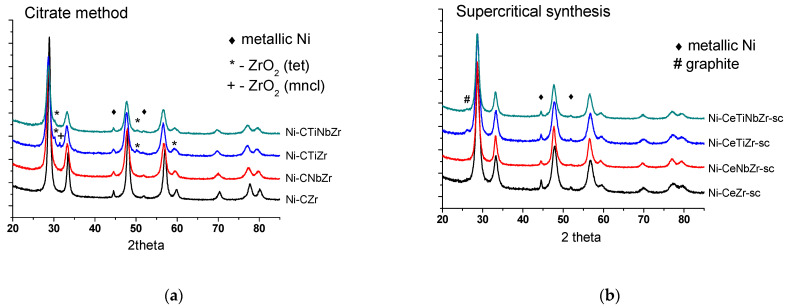
Diffraction patterns of catalysts prepared by citrate methods (**a**) and by supercritical synthesis (**b**) after tests in methane dry reforming.

Diffraction patterns of catalysts after tests in methane dry reforming are presented in Figure 6. The ceria–zirconia mixed oxide structure persists under reaction conditions. Trace amounts of zirconium oxide in citrate samples are due to preparation features and remained unchanged after reaction. The weak peak at 26° corresponding to graphitic carbon (PDF 075-0444) appeared for supercritical catalysts modified by Ti that may be due to a higher content of carbon deposits on their surface.

Fluorite particles size increased for the Ni-CeZr and practically did not change for other citrate samples (Table 2). Hence, it can be suggested that incorporation of titanium and niobium increases stability of oxide particles against sintering. The specific surface area change correlates with the crystallite sizes. It decreased significantly for the unmodified catalyst while remained almost the same for other catalysts or even increased for the sample doped by Ti, which may be due to formation of carbon deposits. Changes in fluorite particle size for all supercritical catalysts are minor.

Nickel oxide is reduced during hydrogen pretreatment with formation of metal particles. Peaks corresponding to Ni^0^ state are observed at 45 and 52°. Nickel metal particle size coincides with that of NiO for Ni-CeNbZr and Ni-CeTiZr compositions, while increased approximately threefold for Ni-CeZr and Ni-CeTiNbZr in both series of catalysts. It suggests that individual Nb or Ti addition prevents nickel particles from sintering. A higher Ni dispersion for samples doped with Ti or Nb correlates with a higher surface Ni content obtained from the XPS data (Section 3.1.2).

It is interesting that Ni particle sizes after the reaction are close in each pair of samples, regardless of the support preparation method and its specific surface area. 

#### 3.3.2. Study of Spent Catalysts by TEM

The morphology of fresh and tested-in-methane dry reforming catalysts was investigated by transmission electron microscopy. Data for fresh catalysts were presented in [30]. Samples are comprised of agglomerated particles with crystallite size ~10 nm for citrate preparation method and 15–20 nm for supercritical synthesis.

**Figure 7:**
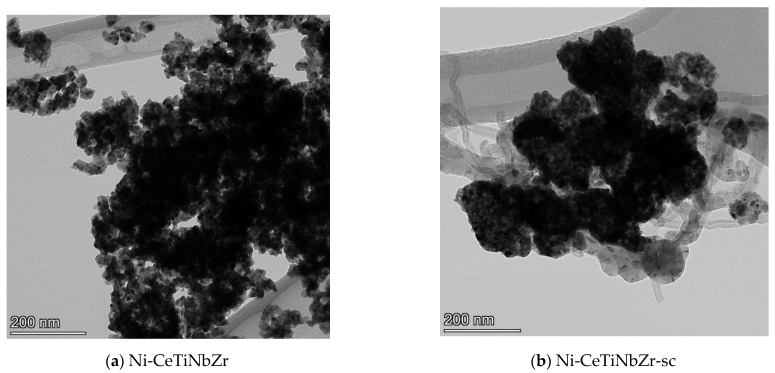
TEM images of Ni-CeTiNbZr catalysts prepared by citrate method (**a**) and by supercritical synthesis (**b**) after testing in methane dry reforming.

Figure 7 shows TEM images of Ni-CeTiNbZr catalysts after tests in methane dry reforming. Images of other catalysts are presented in the Figure S2 in Supplementary Materials. All samples prepared by supercritical synthesis are covered by loose carbon and carbon filaments, while no carbon is observed for catalysts based on supports prepared by citrate method. Some carbon deposits are present only on the Ni-CeNbZr sample (Figure S2).

During hydrogen pretreatment before reaction and subsequently under reaction conditions, nickel oxide is reduced to metallic nickel. For samples prepared by citrate route, nickel particles are in contact with the oxide support (Figure S3a) suggesting strong metal–support interaction. In the case of supercritical synthesis, some metal particles are separated from the support (Figure S3b). This is due to the mechanism of carbon formation during which carbon is diffusing through the Ni particles, and formation of carbon filaments leads to their detachment from the surface [60]. No nickel decoration by oxide support fragments was observed. However, this may be due to the locality of the method.

A significant effect of the support synthesis method on nickel distribution is observed only in the case of Ni-CeTiZr composition (Figure S4). For the citrate method, more uniform and dispersed distribution of nickel is obtained, whereas for supercritical sample there are well-formed metal particles on the catalyst surface. For other samples, a broad particle size distribution is observed. 

#### 3.3.3. Study of Carbon Deposits by TGA

**Figure 8:**
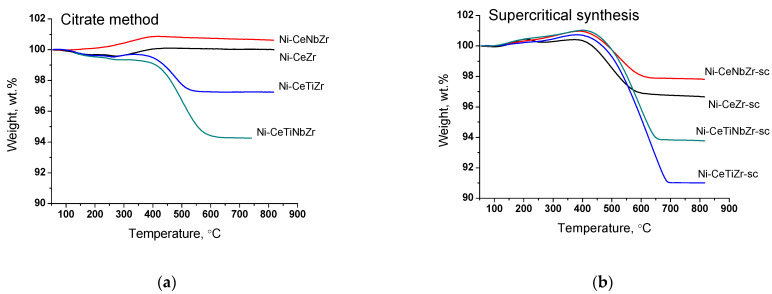
Weight loss during TGA of catalysts prepared by citrate method (**a**) and by supercritical synthesis (**b**) after tests in methane dry reforming.

The amount and nature of coke deposits formed during tests in methane dry reforming were investigated by TGA with mass-spectrometric analysis of effluent gases. The data are presented in the Table 7. Figure 8 presents curves of weight change obtained during TGA. For all samples, the mass increase over 100% at temperature up to 400 °C is observed due to oxidation of metal Ni° to Ni^2+^ and Ce^3+^ to Ce^4+^ [61]. A higher mass increase for catalysts obtained in supercritical conditions may be related to a higher amount of oxygen vacancies (and, thus, Ce^3+^ cations) in their lattice as was shown in our previous work [30] and corresponding ability to take in more oxygen. 

The weight loss of the spent catalysts is associated with the removal of carbon deposits or their precursors (CH_x_ or CH_x_O_y_ species) from the surface. The mass increase for citrate Ni-CeNbZr sample may be observed since there is no removal of carbon deposits, but oxidation of Ni^0^ and Ce^3+^ cations occurs. In both catalysts’ series, the lowest amounts of carbon deposits were obtained for unmodified Ni-CeZr and Ni-CeNbZr compositions. It correlates well with the oxygen diffusion coefficients estimated from the isotope exchange experiments for supercritical catalysts, which were higher for these two compositions and turned out to be significantly lower for titanium-doped samples. 

It was shown that Ti addition leads to a significant increase in the amount of carbon. Similar correlations were also observed in the literature. In the work [62], the authors investigated effect of Ce/Ti ratio on catalytic activity and stability of Ni/CeO_2_–TiO_2_ catalyst and showed that with increasing Ti content the degree of surface carbonization also increases. Similar results were obtained for ethanol steam reforming catalysts in [63]. It was demonstrated in [64] that stability to coking of 5% Ni/Ce_1-x_Ti_x_O_2_ catalysts significantly decreases with increasing Ti amount from 20 to 50% due to decreased (sub)surface lattice oxygen mobility as was shown by transient ^16^O/^18^O isotopic exchange experiments.

At the same time, there is no obvious relation between oxygen diffusion coefficients and catalysts’ activity in the dry reforming of methane in present work. This is probably due to the fact that diffusion of bulk oxygen is sufficiently high for all our ceria–zirconia catalysts and it is not the rate-determining step [65,66]. 

The use of supports prepared in supercritical conditions resulted in formation of a larger quantity of carbon. It is an unexpected result since Ni crystallite sizes obtained from XRD data are close for both catalysts series. The possible explanation is less strong metal–support interaction which resulted in detachment of nickel particles from the oxide support, as was shown by microscopy data, and less effective carbon gasification. 

**Figure 9:**
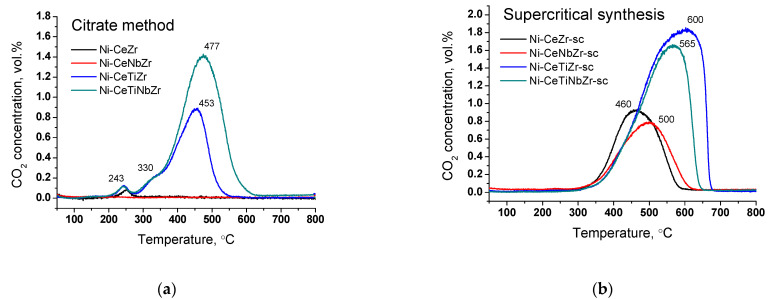
CO_2_ concentration measured by mass-spectroscopy analysis during TGA of catalysts prepared by citrate method (**a**) and by supercritical synthesis (**b**) after tests in methane dry reforming.

It is known that the temperature of coke oxidation depends on its structure. Figure 9 shows change in CO_2_ concentration during oxidation of spent catalysts in TGA experiments. For all citrate samples (except Ni-CeNbZr for which there was no carbon observed), there is a low-temperature peak at ~240 °C corresponding to the removal of amorphous carbon [16,17,18]. 

The shoulder at 330 °C and peak at 450–480 °C for Ti-doped citrate catalysts can be assigned to oxidation of carbon species with a higher degree of graphitization [16,17,18]. All supercritical samples have only one peak of CO_2_ release. Carbon oxidation occurs in the region of 460–500 °C for Ni-CeZr-sc and Ni-CeNbZr-sc samples and shifts towards higher temperatures of 560–600 °C for both catalysts modified with Ti that corresponds to oxidation of graphitic carbon [17,18]. Increased temperature of carbon removal corresponds to increased degree of graphitization of carbon deposits that makes them less prone to oxidation [17].

Summarizing, it was shown that use of supports obtained in supercritical alcohols leads to the formation of more ordered carbon in larger quantities compared to citrate samples, which may be due to stronger metal–support interaction for the last ones, as was shown by microscopy data. For both catalysts’ series, there is no correlation between the carbon amount and nickel crystallite size or XPS results. However, there is a relationship between the amount of carbon deposits and oxygen mobility for the studied samples. For supercritical catalysts, a decreased amount of carbon was obtained for samples with higher oxygen diffusion coefficients, suggesting that for our samples oxygen mobility plays more important role than nickel dispersion.

### 3.4. Long-Term Stability Tests of Catalysts Prepared by Supercritical Synthesis

#### 3.4.1. Long-Term Stability Tests

**Figure 10:**
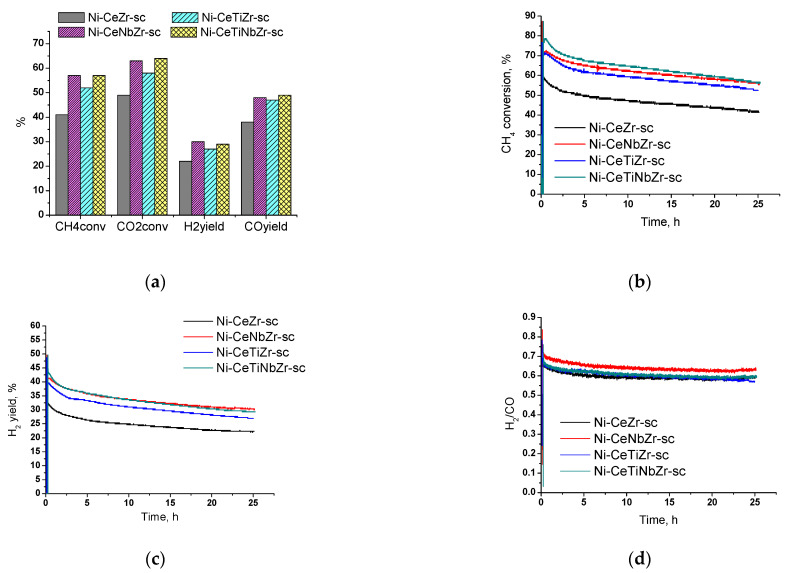
Results of long-term stability tests in methane dry reforming over catalysts prepared by supercritical synthesis: (**a**) values after 25 h of testing, (**b**) methane conversion, (**c**) H_2_ yield, and (**d**) H_2_/CO ratio during testing for 25 h. Reaction feed 15%CH_4_ + 15%CO_2_ + Ar balance.

Since the highest activity in the temperature cycle DRM tests (Section 3.2) was observed for samples prepared in supercritical media, we decided to conduct long-term tests for 25 h to check their stability. The Figure 10a shows the values of reagents conversion and products yield after 25 h of catalyst operation at 700 °C. For all catalysts, the CO_2_ conversion is higher than the CH_4_ conversion due to occurrence of side reverse water–gas shift reaction (Equation (2)).

Catalysts doped with both titanium and niobium showed higher activity compared with the unmodified sample. Methane conversion increases in the row Ni-CeZr-sc < Ni-CeTiZr-sc < Ni-CeNbZr-sc = Ni-CeTiNbZr-sc.

Initial activity increase in the first half-hour of catalyst operation is associated with the establishment of its steady-state (Figure 10b). After reaching the steady-state, sharp activity drop is observed for all compositions in the first three hours. In the remaining time, activity decrease is slower and amounts to 10–13% of methane conversion for all samples. The change in hydrogen yield for all samples has the similar character as methane conversion (Figure 10c). The highest activity at the end of the tests was observed for Ni-CeNbZr-sc and Ni-CeTiNbZr-sc samples. They provided methane conversion of 57% and a hydrogen yield of 30%. The lowest total activity decrease, by 16% in methane conversion, was observed for Ni-CeNbZr-sc sample.

A higher H_2_/CO ratio (Figure 10d) at the beginning of tests may indicate a greater contribution of carbon formation reactions—methane cracking (Equation (3)) and/or Boudouard reaction (Equation (4)). Therefore, it can be suggested, that active carbon formation accompanied by sharp drop in catalytic activity occurs in the first few hours of the DRM reaction; after that, catalysts demonstrate quite stable operation. 

#### 3.4.2. Study of Carbon Deposits after Long-Term Tests by TGA

**Figure 11:**
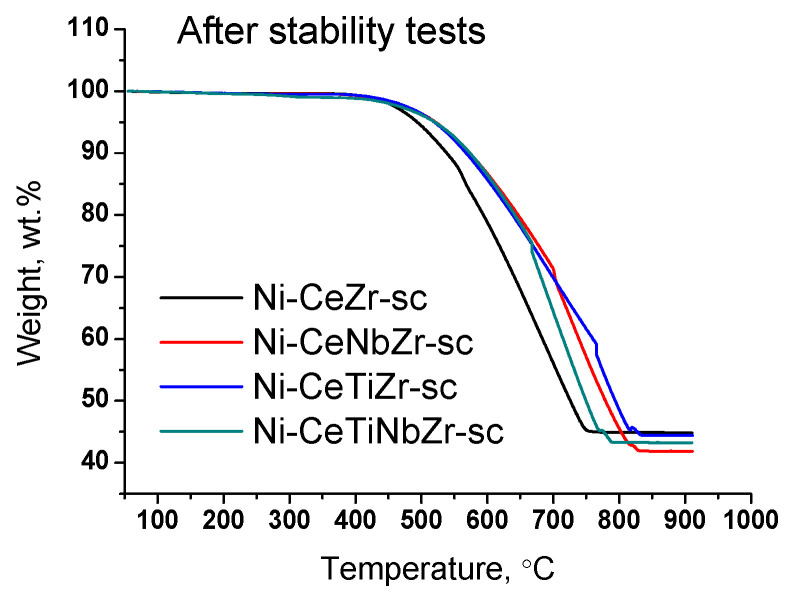
Weight loss during TGA of catalysts prepared by supercritical synthesis after long-term stability tests in methane dry reforming.

**Figure 12:**
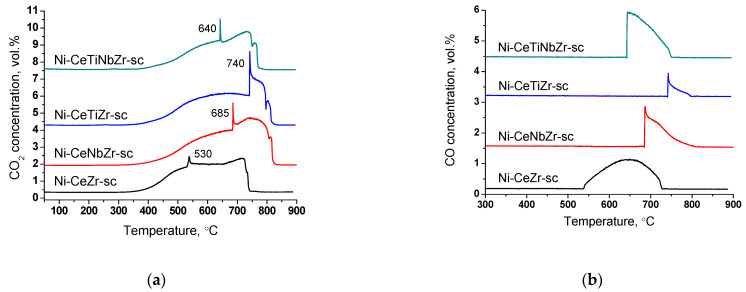
CO_2_ (**a**) and CO (**b**) concentrations measured by mass-spectroscopy analysis during TGA of catalysts prepared by supercritical synthesis after long-term stability tests in methane dry reforming.

Results of TGA experiments of catalysts after long-term stability tests in DRM are presented in Figure 11 and Figure 12, and in Table 7. There are several peaks of CO_2_ release (Figure 12a) indicating the existence of various types of carbon. For all samples, CO_2_ formation begins at higher temperatures than for catalysts tested in short-term experiments (Section 3.3.3) suggesting higher degree of carbon graphitization. In general, in the temperature range 350–650 °C graphitic carbon is removed [16,17,18]. The catalyst Ni-CeTiNbZr-sc that showed the highest activity during long-term stability tests was also examined by microscopy (Figure S6). The presence of several forms of carbon has been shown—carbon layers, including those with encapsulated metal particles, and carbon fibers with various defects. It should be noted that defect carbon fibers are oxidized at lower temperature than the normal ones. There are also some metallic particles in contact with the oxide support which are free from carbon. 

At higher temperatures, a sharp increase in CO_2_ concentration accompanied by CO appearance (Figure 12b) in products and a sharp mass drop on the TGA curve is observed (Figure 11). Such a spike-shape of the peak is observed due to sharp increase in CO_2_ formation rate and insufficient recording rate of detector to obtain usual sloping peak. The combustion of carbon is a complex topochemical reaction, the rate of which passes through a maximum with its transformation. The sharp increase in carbon burning rate can be connected with several factors: release of support surface from amorphous carbon and participation of support oxygen in the gasification of carbon deposits; oxidation of metallic nickel to nickel oxide and more efficient combustion of carbon on its surface; process of carbon self-ignition, which can occur at different temperatures for various forms of carbon. The appearance of CO is associated with incomplete oxidation of carbon due to oxygen lack.

High temperature peaks in the 650–850 °C temperature range correspond to the removal of whisker type carbon [16,18]. 

For doped samples, the mass drop ends at higher temperature and the rate of weight loss is lower (Figure 11), which suggests a higher degree of graphitization of carbon deposits [17,67]. 

It is known that the filamentous carbon does not block the catalysts’ surface, therefore its formation does not lead to catalyst deactivation in the first catalytic cycle [18,67,68]. In the work [68], the 5% Ni/SiO_2_ catalyst showed a stable operation in DRM reaction for 10 h at 800 °C, although the weight loss of spent catalysts corresponding to removal of the whisker carbon was 65%. In the work [67], the La-NiMgAlO catalyst was tested in DRM for 200 h at various temperatures—650, 700 and 750 °C. Contrary to expectations, the catalyst tested at 750 °C, which showed the best stability, exhibited the highest weight loss of about 70% and large amount of carbon fibers. For catalysts tested at lower temperatures, the formation of coating carbon was observed in a greater extent, what led to their deactivation.

At temperatures above 600 °C, carbonate removal can also contribute to weight loss [18]. However, the CO_2_ release occurred synchronously with the O_2_ consumption (Figure S5), indicating that CO_2_ is formed mainly due to the oxidation of carbon deposits.

Significant weight loss from 55 to 58% is observed for all samples (Table 7). Thus, we can conclude that in the case of long-term tests at 700 °C the introduction of the dopant does not affect the amount of carbon deposits, but strongly affect the catalyst activity.

In the work [17], the authors compared the effect of La and Ce addition on stability of 8%Ni/ZrO_2_ to carbon formation in DRM at various reaction temperatures 550–800 °C. It was shown that addition of promoter leads to increased activity and amount of carbon at low temperatures. However, the difference in the carbon amount decreases with increasing temperature. In addition, the influence of La and Ce on catalytic activity was found to be close.

In our case, reaction temperature is supposed to be high enough so that the effect of the doping cation on the amount of carbon deposits is not observed. 

**Figure 13:**
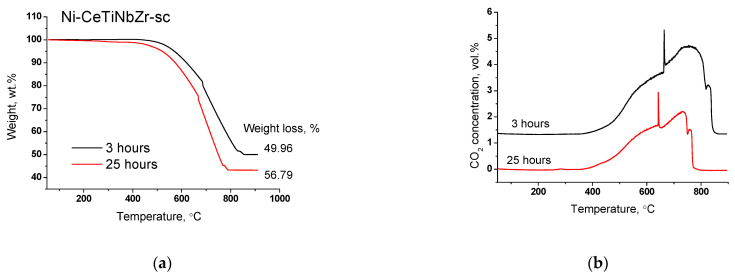
Weight loss (**a**) and CO_2_ concentration (**b**) during TGA of Ni-CeTiNbZr-sc after long-term stability tests for 25 and 3 h in methane dry reforming.

The most active catalyst Ni-CeTiNbZr-sc was additionally tested in DRM for three hours to study the amount and nature of carbon deposits. The TGA results and mass-spectroscopy data are presented in the Figure 13. The amount of carbon after three hours of testing was found to be close to that after 25 h of operation. CO_2_ evolution begins at the same temperature and ends at 60 degrees higher temperature that suggests a higher graphitization degree of carbon deposits. Thus, we confirmed the assumption that the main deactivation occurs in the first hours of catalyst operation.

It is known that the carbon formation takes place mainly on large ensembles of nickel [35]. TGA data indicate the presence of graphitic carbon, which is capable to block the catalyst surface. So, it can be assumed that in the first hours of the reaction, carbon is formed on the most active centers and then blocks them, due to which a sharp drop in activity is observed. After that, the catalyst reaches a steady-state and the subsequent activity decrease occurs not due to the carbon formation, but due to the sintering of the active component. However, this assumption requires further research. 

## 4. Conclusions

Nickel-containing catalysts based on fluorite-like supports prepared by the citrate method and by original supercritical synthesis were studied before and after tests in DRM reaction. According to the XPS data, the preparation method has a strong influence on the surface composition. Catalysts prepared by supercritical synthesis contain much more cerium ions on the surface than catalysts synthesized by the citrate method. The number of Ni species is also higher for catalysts prepared by supercritical synthesis. No CO adsorption bands were observed at room temperature in the FTIR spectra of adsorbed CO on the doped citrate samples. 

The oxygen mobility of supercritical samples was studied by the temperature-programmed isotope exchange (TPIE) with C^18^O_2_. Incorporation of titanium results in almost fourfold decrease in oxygen mobility compared to undoped ceria–zirconia. The effect of nickel consists in decrease in the oxygen diffusion coefficients by 2–5 times and is probably associated with a decrease in the oxygen permeability of the near-surface layers of catalysts. For spent catalysts doped by Ti and Nb, metal Ni particles are of the same size as the initial NiO (~25 nm), suggesting that incorporation of these cations prevents nickel particles from sintering regardless of the preparation method. 

The catalysts after tests in DRM were investigated by TGA coupled with temperature-programmed oxidation with mass-spectrometric analysis of effluent gases. After short-term temperature tests, the lowest amounts of carbon deposits were obtained for unmodified Ni-CeZr and Ni-CeNbZr compositions (lack of carbon for citrate samples and 2–3% for supercritical ones) that correlates well with their higher oxygen mobility estimated for supercritical catalysts. No correlation between the carbon amount and nickel crystallite size or XPS results is observed. The use of supports obtained in supercritical alcohols leads to formation of more ordered carbon in larger quantities (max. ~9%) compared to citrate samples that may be connected with stronger metal–support interaction for the last ones, which was shown by microscopy data. 

Catalysts prepared by supercritical synthesis were tested in DRM for 25 h to check their stability. The highest activity drop due to carbon formation was observed in the first three hours. In the remaining time, catalysts demonstrate quite stable operation and activity decrease of 10–13% of methane conversion for all samples. The highest activity was observed for Ni-CeNbZr-sc and Ni-CeTiNbZr-sc samples, corresponding to methane conversion of 57% and a hydrogen yield of 30% at the end of tests. For all compositions, close values of weight loss (~55%) were observed. So, in the case of long-term tests at 700 °C, the introduction of the dopant does not affect the amount of carbon deposits, but strongly affect the catalyst activity. 

## Figures and Tables

**Table 1:** Catalysts’ compositions and abbreviations.

№	Composition	Synthesis Method	
		Citrate	Supercritical
1	5%Ni/Ce_0.75_Zr_0.25_O_2-δ_	Ni-CeZr	Ni-CeZr-sc
2	5%Ni/Ce_0.75_Nb_0.1_Zr_0.15_O_2-δ_	Ni-CeNbZr	Ni-CeNbZr-sc
3	5%Ni/Ce_0.75_Ti_0.1_Zr_0.15_O_2-δ_	Ni-CeTiZr	Ni-CeTiZr-sc
4	5%Ni/Ce_0.75_Ti_0.05_Nb_0.05_Zr_0.15_O_2-δ_	Ni-CeTiNbZr	Ni-CeTiNbZr-sc

**Table 2:** Textural and structural properties of fresh and spent catalysts.

Sample	Fresh				After Reaction			
	S_BET_, m^2^/g	V_total_, cm³/g	d sup *, nm	d NiO **, nm	S_BET_, m^2^/g	V_total_, cm³/g	d sup *, nm	d Ni **, nm
**Citrate method**								
Ni-CeZr	35	0.14	11	18	22	0.15	17	65
Ni-CeNbZr	29	0.11	11	24	28	0.13	11	23
Ni-CeTiZr	26	0.19	11	24	36	0.19	11	24
Ni-CeTiNbZr	28	0.21	11	18	30	0.24	11	52
**Supercritical synthesis**								
Ni-CeZr-sc	21	0.14	9	30	11	0.19	11	77
Ni-CeNbZr-sc	17	0.19	14	28	15	0.29	13	26
Ni-CeTiZr-sc	23	0.18	10	20	24	0.17	11	25
Ni-CeTiNbZr-sc	26	0.18	15	24	14	0.15	12	50

* d sup—crystallite size of fluorite phase according to XRD data. ** d NiO and d Ni—crystallite sizes according to XRD data.

**Table 3:** Atomic ratios of elements in the (sub)surface layers of catalysts after reduction treatment in H_2_ at 450 °C.

Sample	[Ce]*	[Ce^3+^]/[Ce], %	[Ni] *	[Ni^2+^]/[Ni], %	[Ti] *	[Nb] *
**Citrate method**						
Ni-CeZr	0.70	59	0.08	12	-	-
Ni-CeNbZr	0.63	71	0.11	7	-	0.20
Ni-CeTiZr	0.74	62	0.10	10	0.08	-
Ni-CeTiNbZr	0.70	61	0.08	10	0.03	0.13
**Supercritical synthesis**						
Ni-CeZr-sc	0.81	50	0.15	10	-	-
Ni-CeNbZr-sc	0.79	60	0.15	10	-	0.13
Ni-CeTiZr-sc	0.79	55	0.19	10	0.10	-
Ni-CeTiNbZr-sc	0.75	60	0.10	10	0.04	0.09

* the ratio is normalized to [Ce] + [Zr] + [Ti] + [Nb].

**Table 4:** Concentration of various type of surface carbonyls according to IR spectroscopy.

Sample	νCO, cm^−^	Sites Amount, μmol/g	Bridging CO Amount, μmol/g	On-top CO Amount, μmol/g	Total Amount, μmol/g
Ni-CeZr	1905 1954 1980 2027 2063	14 2 16 2 12	32	14	46
Ni-CeZr-sc	1839 1937 1955 1975 2047 2055 2080	0.1 17 4 3 9 2 5	24	16	40
Ni-CeNbZr-sc	1903 1953 2040 2057 2076	5 6 8 2 5	11	15	26
Ni-CeTiZr-sc	1917 1954 2032 2067 2079	8 1 7 4 0.3	9	11	20
Ni-CeTiNbZr-sc	1933 1949 2039 2067 2083	15 0.5 8 7 2	16	17	33

**Table 5:** Weighted average oxygen tracer diffusion coefficients for samples at 600 °C and their effective activation energies according to TPIE with C^18^O_2_ data.

Sample		S m^2^/g	L, 10^−6^ cm	(D/L^2^)_over_, 10^−4^ s^−1^	D_over_, 10^−16^ cm^2^/s	E kJ/mol	β
**Citrate method**							
CeZr	oxide	44	0.9	52	42	75	38
**Supercritical synthesis**							
CeZr-sc	oxide	29	1.5	22	43	75	13
	Ni/oxide	21	2.0	5.9	24	77	9
CeNbZr-sc	oxide	20	2.2	10	48	75	7
	Ni/oxide	16	2.7	2.0	14	75	4
CeTiZr-sc	oxide	28	1.6	4.9	12	75	33
	Ni/oxide	23	1.9	2.8	9.7	85	14
CeTiNbZr-sc	oxide	22	2.0	6	24	75	29
	Ni/oxide	26	1.7	1.8	5.2	83	9

**Table 6:** Steady-state CH_4_ conversions during methane dry reforming at different temperatures.

Sample	Temperature, °C						
	600	650	700	750	700	650	600
**Citrate method**							
Ni-CeZr	17	27	37	48	33	22	13
Ni-CeNbZr	17	26	35	43	29	19	10
Ni-CeTiZr	17	27	36	43	29	18	10
Ni-CeTiNbZr	0	17	26	35	25	16	9
**Supercritical synthesis**							
Ni-CeZr-sc	26	29	35	38	29	19	11
Ni-CeNbZr-sc	11	19	23	30	20	13	7
Ni-CeTiZr-sc	13	20	27	33	24	16	10
Ni-CeTiNbZr-sc	25	35	44	51	38	27	17

**Table 7:** Weight loss during TGA of spent catalysts.

Sample	Mass Loss, %TGA	
	Temperature Tests	Long-Term Tests
**Citrate method**		
Ni-CeZr	0	-
Ni-CeNbZr	0	-
Ni-CeTiZr	2.75	-
Ni-CeTiNbZr	5.80	-
**Supercritical synthesis**		
Ni-CeZr-sc	3.34	55.20
Ni-CeNbZr-sc	2.19	58.13
Ni-CeTiZr-sc	8.99	55.61
Ni-CeTiNbZr-sc	6.24	56.79

## Data Availability

Not applicable.

## Supplementary Materials

Study of oxygen mobility by isotope exchange with C18O2. Figures S1–S6. The following supporting information can be downloaded at: https://www.mdpi.com/article/10.3390/nano12203676/s1.
