# Supplementary material for: Carbon Formation during Methane Dry Reforming over Ni-Containing Ceria-Zirconia Catalysts
Source: Nanomaterials (Basel). 2022 Oct 19;12(20):3676. doi: 10.3390/nano12203676 (PMC9607641; doi:10.3390/nano12203676)
Supplement: Supplementary file 1 [file nanomaterials-12-03676-s001.zip › nanomaterials-1955672-supplementary.pdf]

Supplementary Information:

Carbon Formation during Methane Dry Reforming over Ni-Containing Ceria-Zirconia Catalysts

Ekaterina Smal, Yulia Bespalko, Marina Arapova, Valeria Fedorova, Konstantin Valeev, Nikita Eremeev,
Ekaterina Sadovskaya, Tamara Krieger, Tatiana Glazneva, Vladislav Sadykov and Mikhail Simonov *

Department of Heterogeneous Catalysis, Boreskov Institute of Catalysis,
630090 Novosibirsk, Russia
* Correspondence: smike@catalysis.ru

Supplement A. Study of oxygen mobility by isotope exchange with $C^{18}O_2$

^{18}O atomic fraction (α) and $C^{16}O^{18}O$ molecular fraction ($f_{16\text{-}18}$) were calculated as follows:

$$\alpha = \frac{I_{48} + 0.5 \times I_{46}}{I_{44} + I_{46} + I_{48}}, \quad f_{16\text{-}18} = \frac{I_{46}}{I_{44} + I_{46} + I_{48}}. \qquad \text{(S1)}$$

Here I_{44}, I_{46} and I_{48} are parent peaks at m/e = 44, 46 and 48, respectively.

The analysis α and $f_{16\text{-}18}$ responses was made for calculating the values of the heteroexchange rate (R), oxygen tracer diffusion coefficient (D^*) and their effective activation energies ($E_{a,R}$, $E_{a,D}$) using a mathematical model:

$$\frac{\partial \alpha_g}{\partial t} + O(\tau) = \frac{N_s}{N_g} R^{\Sigma}(\alpha_s - \alpha_g), \qquad \text{(S2)}$$

$$\frac{\partial \alpha_s}{\partial t} = R^{\Sigma}(\alpha_g - \alpha_s) - \frac{N_{bulk}}{N_s} \frac{D^*}{L^2} \left.\frac{\partial \alpha_{bulk}}{\partial \eta}\right|_{\eta=0}, \qquad \text{(S3)}$$

$$\frac{\partial \alpha_{bulk}}{\partial t} = \frac{D^*}{L^2} \frac{\partial^2 \alpha_{bulk}}{\partial \eta^2}, \qquad \text{(S4)}$$

$$\frac{\partial f_{16-18}}{\partial t} + O(\tau) = R^0(2\alpha_g(1 - \alpha_g) - f_{16-18}) + \frac{N_s}{N_g}(R^2(2\alpha_s(1 - \alpha_s) - f_{16-18}) + R^1(\alpha_g(1 - \alpha_s) + \alpha_s(1 - \alpha_g) - f_{16-18})). \qquad \text{(S5)}$$

Initial and boundary conditions are: $t = 0 \Rightarrow \alpha_i = \alpha_i^0$.

In the equations (A2)–(A5), α_g, α_s, and α_{bulk} are the ^{18}O atomic fractions in the gas-phase reactant, at the sample surface, and in the sample bulk, respectively; N_g, N_s, and N_{bulk} are the number of oxygen atoms in the gas phase, at the oxide surface, and in the oxide bulk, respectively; f_{16-18} is the $C^{16}O^{18}O$ molecular fraction; R^0, R^1, and R^2 are the rates of the exchange different types, in the classification of Muzykantov; $R^{\Sigma} = 0.5R^1 + R^2$ is the total heteroexchange summary rate, D^* is the

oxygen tracer diffusion coefficient, L is the sample particle characteristic size, η is the dimensionless distance to the sample surface, t is the time, $O(\tau)$ is the operator dependent on the mass transport regime in the reactor:

$$O(\tau) = \frac{1}{\tau}\frac{\partial \alpha_g \ (\text{or } f_{16-18})}{\partial \xi}. \qquad \text{(S6)}$$

Here τ is the contact time in the reactor, ξ is the catalyst layer dimensionless length.

The exchange rates and diffusion coefficients are expressed by the Arrhenius-type dependences:

$$R^{(i)} = R^{(i)}_{\text{ref}} \exp\left(-\frac{E_{a,R(i)}}{RT'}\right), \quad D^* = D_{\text{ref}} \exp\left(-\frac{E_{a,D}}{RT'}\right), \quad T' = \frac{TT_{\text{ref}}}{T_{\text{ref}} - T}, \qquad \text{(S7)}$$

where $R^{(i)}_{\text{ref}}$ and D_{ref} are the exchange rate and diffusion coefficient at a temperature T_{ref}, respectively; $E_{a,R(i)}$ and $E_{a,D}$ are their apparent activation energies.

Average tracer diffusion coefficient (D^*_{over}) values were used for comparison of oxygen mobility:

$$D^*_{over} = \sum_i \frac{\theta_i \times D^*_i}{100\%}, \qquad \text{(S8)}$$

where θ_i is the fraction of oxide anions characterized by a tracer diffusion coefficient D^*_i.

A nonuniformity coefficient (β) determined as a ratio of the average tracer diffusion coefficient of 50 % fraction of oxygen substituting relatively fast to the average tracer diffusion coefficient of the rest fraction of oxygen:

$$\beta = \frac{\sum_{i,\,fast} \theta_i \times D^*_i}{\sum_{j,slow} \theta_j \times D^*_j}. \qquad \text{(S9)}$$

Supplement B.

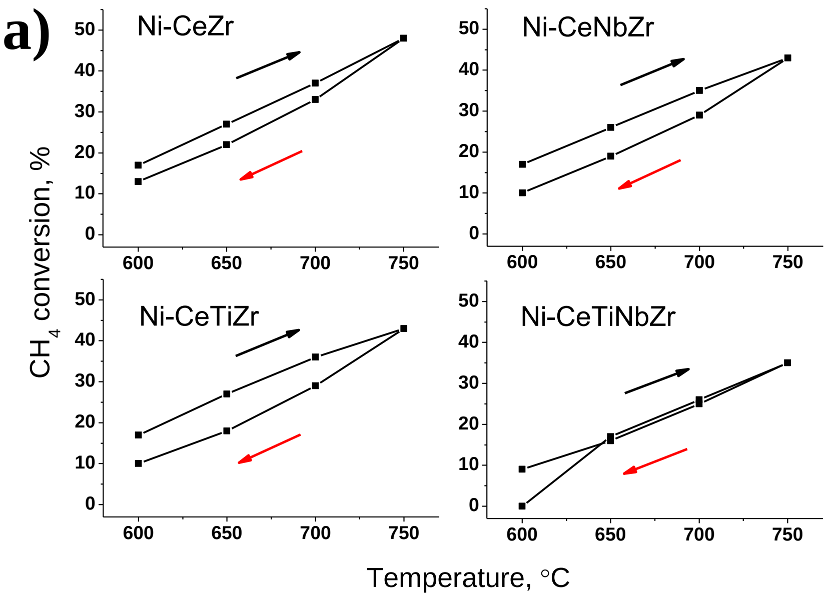

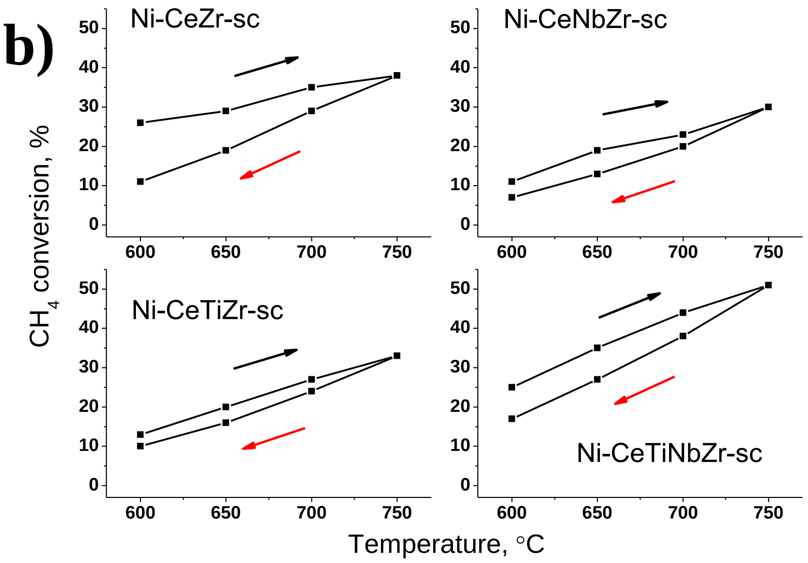

Figure S1. Steady-state CH_4 conversions during methane dry reforming at different temperatures over catalysts prepared by citrate method (a) and by supercritical synthesis (b). Reaction feed 15%CH_4 + 15%CO_2 + N_2 balance, contact time 10 ms. Black arrow - temperature rise, red arrow - temperature drop.

Ni-CeZr	Ni-CeZr-sc

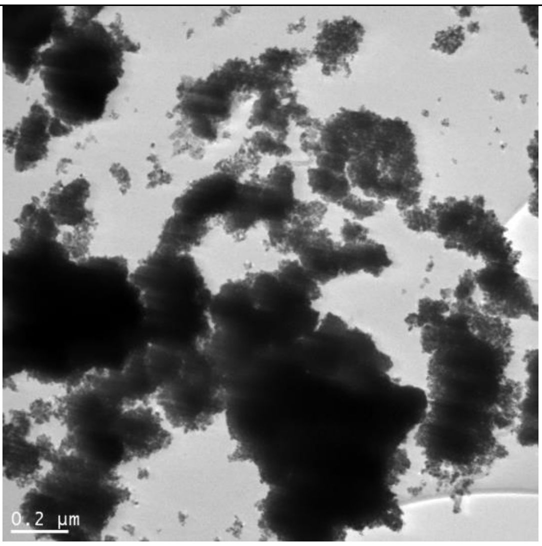 a)	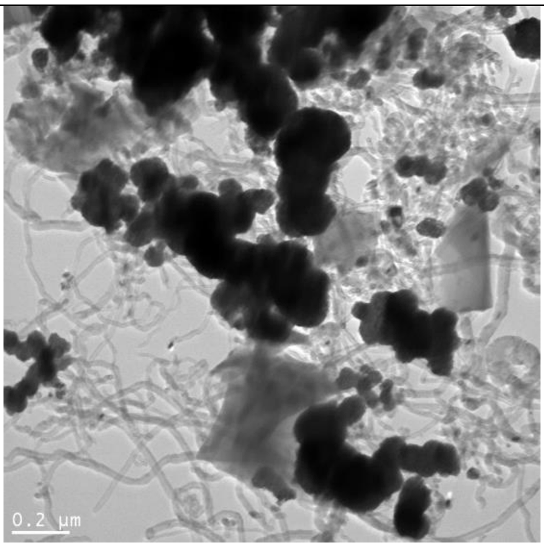 b)
Ni-CeNbZr 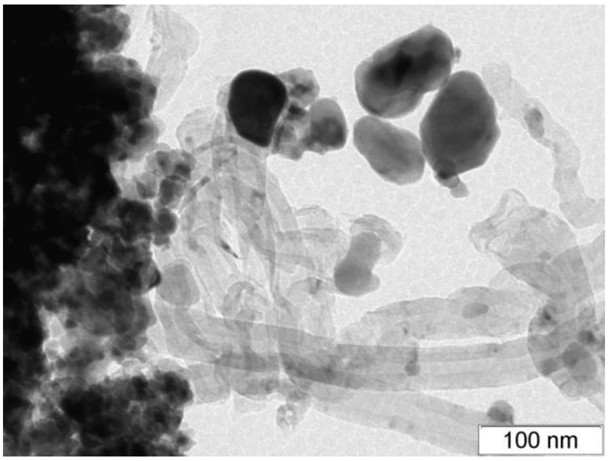 c)	Ni-CeNbZr-sc 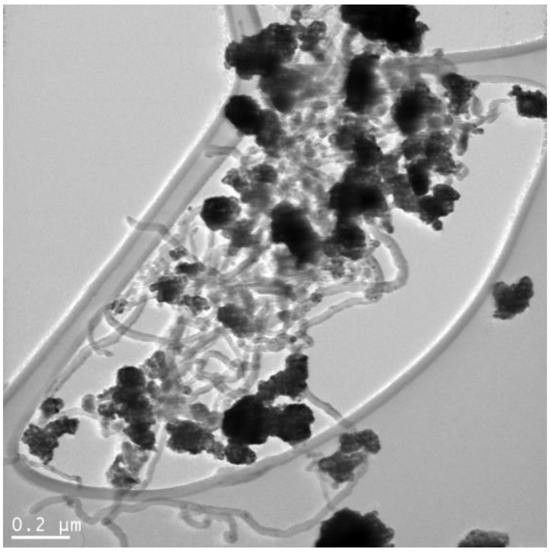 d)
Ni-CeTiZr 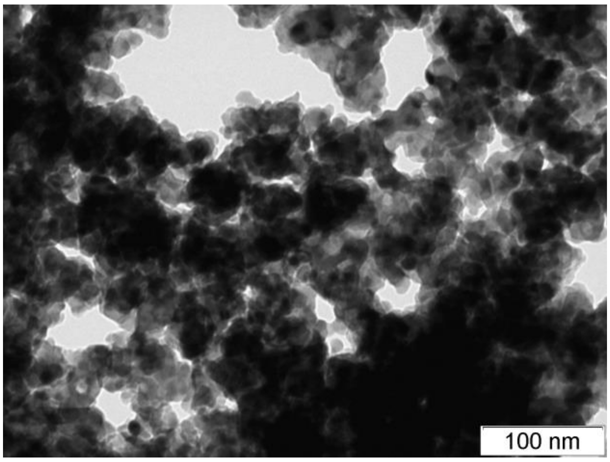 e)	Ni-CeTiZr-sc 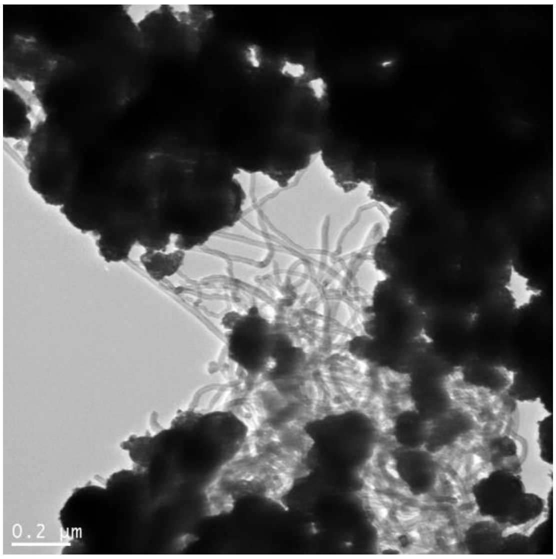 f)

Figure S2. TEM images of catalysts prepared by citrate method (left column) (a) Ni-CeZr, c) Ni-CeNbZr, e) Ni-CeTiZr) and by supercritical synthesis (right column) (b) Ni-CeZr-sc, d) Ni-CeNbZr-sc, f) Ni-CeTiZr-sc) after testing in methane dry reforming.

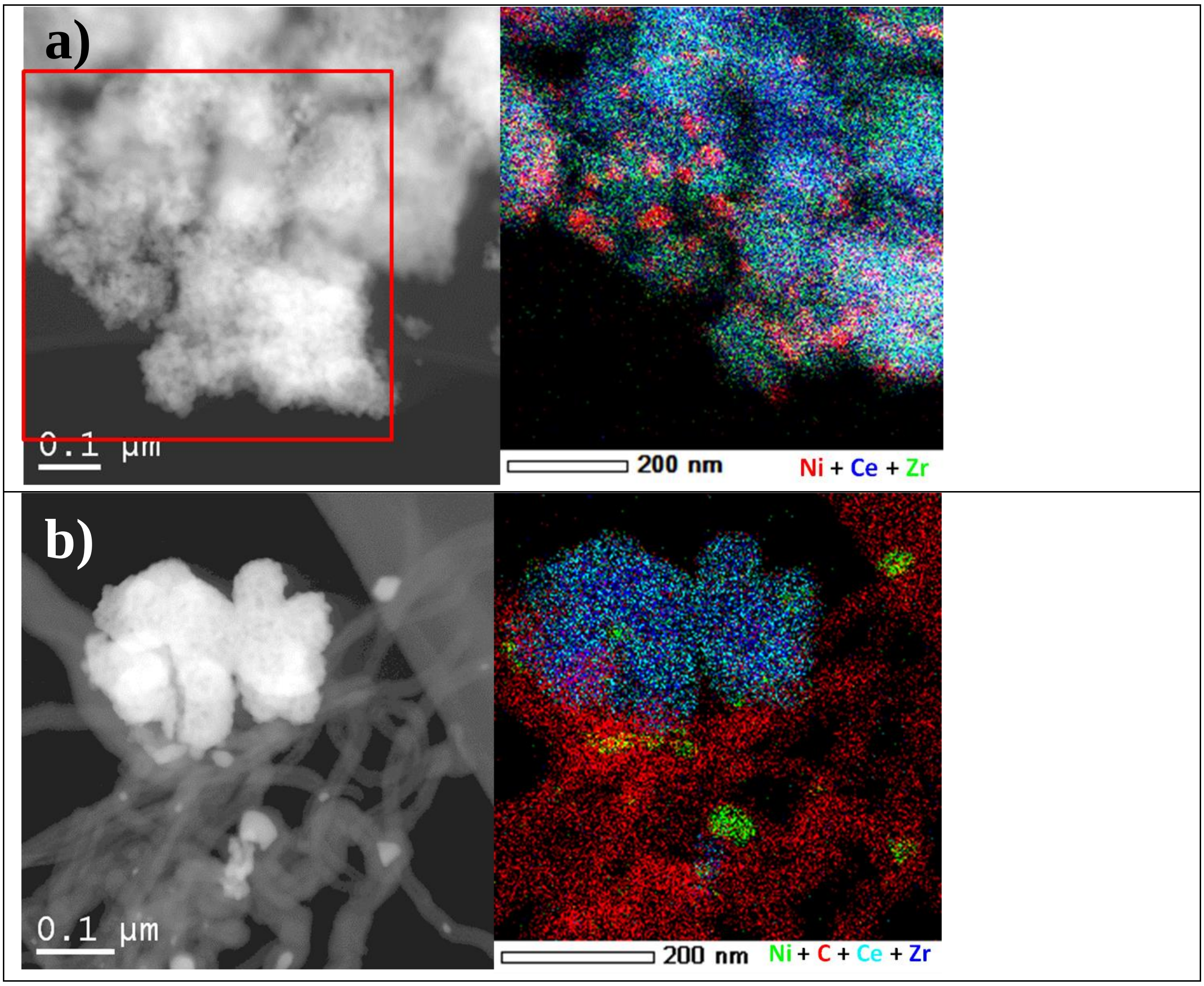

Figure S3. TEM images of Ni-CeZr catalyst prepared by citrate method (a) and by supercritical synthesis (b) after testing in methane dry reforming. Red square at the left subfigure a) corresponds to the area of EDX analysis at the right one.

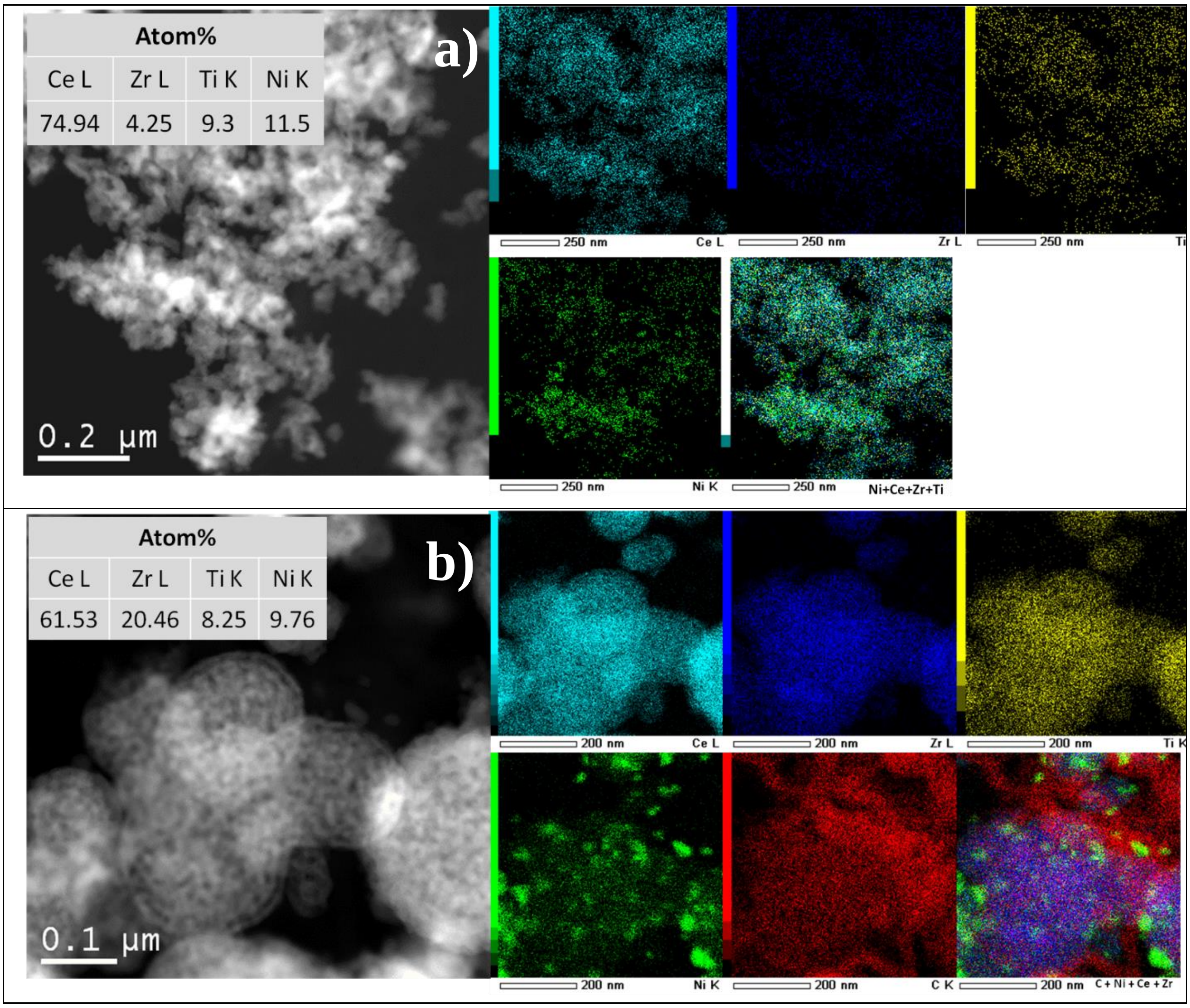

Figure S4. TEM images of Ni-CeTiZr catalyst prepared by citrate method (a) and by supercritical synthesis (b) after testing in methane dry reforming.

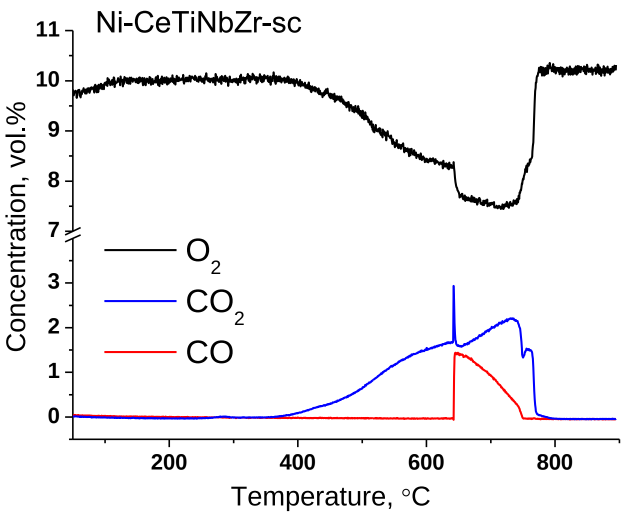

Figure S5. Data of mass-spectroscopy analysis during TGA of Ni-CeTiNbZr-sc after long-term stability tests in methane dry reforming.

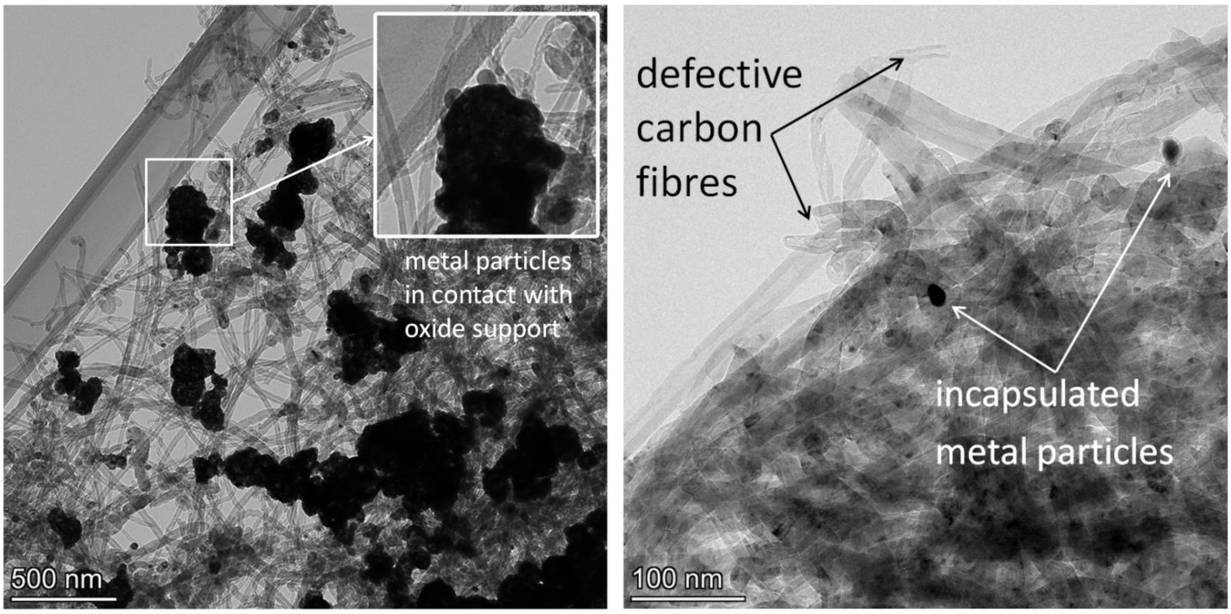

Figure S6. TEM images of Ni-CeTiNbZr-sc catalyst after long-term stability tests in methane dry reforming.
